# Etiology of Maculopapular Rash in Measles and Rubella Suspected Patients from Belarus

**DOI:** 10.1371/journal.pone.0111541

**Published:** 2014-10-30

**Authors:** Marina A. Yermalovich, Galina V. Semeiko, Elena O. Samoilovich, Ekaterina Y. Svirchevskaya, Claude P. Muller, Judith M. Hübschen

**Affiliations:** 1 Republican Research and Practical Center for Epidemiology and Microbiology, Minsk, Belarus; 2 Institute of Immunology, Centre de Recherche Public de la Santé/Laboratoire National de Santé, Luxembourg, Luxembourg; Columbia University, United States of America

## Abstract

As a result of successful implementation of the measles/rubella elimination program, the etiology of more and more double negative cases remains elusive. The present study determined the role of different viruses as causative agents in measles or rubella suspected cases in Belarus. A total of 856 sera sent to the WHO National Laboratory between 2009 and 2011 were tested for specific IgM antibodies to measles virus (MV), rubella virus (RV) and human parvovirus B19 (B19V). The negatives were further investigated for antibodies to enterovirus (EV) and adenovirus (AdV). Children of up to 3 years were tested for IgM antibodies to human herpesvirus 6 (HHV6). A viral etiology was identified in 451 (52.7%) cases, with 6.1% of the samples being positive for MV; 2.6% for RV; 26.2% for B19V; 9.7% for EV; 4.6% for AdV; and 3.6% for HHV6. Almost all measles and rubella cases occurred during limited outbreaks in 2011 and nearly all patients were at least 15 years old. B19V, EV and AdV infections were prevalent both in children and adults and were found throughout the 3 years. B19V occurred mainly in 3–10 years old children and 20–29 years old adults. EV infection was most common in children up to 6 years of age and AdV was confirmed mainly in 3–6 years old children. HHV6 infection was mostly detected in 6–11 months old infants. Laboratory investigation of measles/rubella suspected cases also for B19V, EV, AdV and HHV6 allows diagnosing more than half of all cases, thus strengthening rash/fever disease surveillance in Belarus.

## Introduction

Laboratory confirmation of measles and rubella started in Belarus in 2002. Ever since, serum samples from all suspected cases are investigated at the WHO National Laboratory for Measles and Rubella. An effective vaccination strategy starting in 1967 with monovalent measles vaccine and in 1996 with combined measles/mumps/rubella vaccine has reduced incidence rates for both infections to very low levels. Since 2007 only single imported or importation-related cases have been reported [1].

During the elimination stage WHO recommends that countries demonstrate a sensitivity of their surveillance system of at least 2 suspected and discarded measles/rubella cases per 100 000 population per year [2]. With a decrease of measles and rubella incidence, the etiology of increasing numbers of double negative cases remains unknown. The confirmation of another infectious etiology would be of clinical interest, support the rejection of measles or rubella negative cases and improve the commitment of medical staff to report measles/rubella suspected patients.

Besides bacterial infections, allergic and autoimmune reactions, viral infections are among the more common causes of acute exanthematous diseases [3]. The prevalence of rash/fever agents differs depending on the geographic region, the age groups involved and other epidemiological factors [4]–[6]. The present study determined the incidence of measles virus (MV), rubella virus (RV), human parvovirus B19 (B19V), enterovirus (EV), adenovirus (AdV), and human herpesvirus 6 (HHV6) in measles or rubella suspected patients in Belarus.

## Materials and Methods

### Ethics statement

The serum samples were collected by medical staff in hospitals or outpatient clinics for diagnostic purposes in the framework of the Belarus measles/rubella surveillance. According to the National Public Health Law in Belarus, taking a blood sample for diagnostic purposes requires only verbal informed consent. This consent was obtained from all measles/rubella suspected patients investigated here or from their guardians. The fact that a blood sample has been taken is entered in the medical records of the patient by the treating physician. Upon arrival at the WHO National Measles and Rubella Reference Laboratory, a unique identifier number was attributed to each sample and was used for all data analyses. Access to patient data including name, date of birth, gender, place of residence, vaccination status, date of rash onset and sample collection was restricted to people directly involved in diagnosis and reporting to the treating physician.

### Study samples

Between January 2009 and December 2011 a total of 856 serum samples from measles/rubella suspected patients were tested at the WHO National Laboratory of Belarus. With 276 samples in 2009, 190 in 2010 and 390 in 2011, case investigation rates per 100 000 population were 2.9, 2.0 and 4.1. The patients were between less than 1 and 61 years old and were from all over the country.

### Laboratory testing

All samples were tested for specific IgM antibodies to MV, RV and B19V. The sera that were negative for all three pathogens (n = 559) were further tested for specific IgM antibodies to EV and AdV. From 156 children up to 3 years of age samples were available for HHV6 testing.

Virus-specific IgM antibodies to MV, RV, B19V, EV and AdV were investigated using commercial ELISA kits (Enzygnost anti-measles and anti-rubella virus IgM, Siemens, Germany; Parvovirus B19 IgM and Enterovirus IgM, Virion/Serion, Germany; Adenovirus IgM ELISA, DRG, Germany). IgM antibodies to HHV6 were detected using an indirect immunofluorescence kit (Anti-HHV-6 IIF IgM, Euroimmun, Germany) according to the manufacturers' recommendations.

### Statistical analysis

Statistical analyses were done with the Fisher's exact test using StatSoft STATISTICA v.8.0 software.

## Results

Overall a viral etiology was identified in 451 of the 856 measles/rubella suspected cases (52.7%) with the highest detection rate in 2011 and the lowest in 2009 (Table 1). Positives were found for all six investigated pathogens and B19V, EV, AdV and HHV6 were detected in each of the 3 consecutive years (Table 1).

**Table 1:** Number of sera tested and IgM antibody positives for measles virus (MV), rubella virus (RV), human parvovirus B19 (B19V), enterovirus (EV), adenovirus (AdV) and human herpesvirus 6 (HHV6) infections, Belarus, 2009–2011.

Year	No. tested for MV, RV, B19V	Number (%) of positives for			No. tested for EV, AdV*	Number (%) of positives for		No. tested for HHV6**	Number (%) of positives for	Total positive No. (%)
		MV	RV	B19V		EV	AdV		HHV6	
**2009**	276	0	2 (0.7)	81 (29.4)	193	33 (17.1)	7 (3.6)	46	9 (19.6)	132 (47.8)
**2010**	190	1 (0.5)	0	59 (31.1)	130	24 (18.5)	3 (2.3)	36	7 (19.4)	94 (49.5)
**2011**	390	51 (13.1)	20 (5.1)	84 (21.5)	235	26 (11.1)	29 (12.3)	74	15 (20.3)	225 (57.7)
**Total**	856	52 (6.1)	22 (2.6)	224 (26.2)	558	83 (14.9)	39 (7.0)	156	31 (19.9)	451 (52.7)

*MV/RV/B19V negatives were tested.

**only children up to 3 years of age were tested.

Between 2009 and 2011 a total of 6.1% and 2.6% of the samples were positive for measles and rubella, respectively, with significant differences between the years (p = 0.001). A single measles case was found in 2009/2010, but in 2011 51 cases or 13.1% of all samples were positive for measles and this was the most frequent etiology after B19V in that year (Table 1). Only 2 cases of rubella were detected before 2011 when 20 cases were confirmed. Despite some fluctuation in incidence rates (21.5–31.1%), B19V was consistently the main etiology of measles/rubella suspected cases (Table 1). 9.7% of all samples or 14.9% of the 558 samples tested were positive for EV with the highest rates in 2009 and 2010. AdV infection was confirmed in 4.6% of all cases or 7.0% of the 558 samples tested with the highest rate in 2011 (Table 1). HHV6 specific antibodies were detected in 31 samples corresponding to 3.6% of all measles/rubella suspected cases or 19.9% of the 156 samples tested, with essentially no differences between the years (p = 1.0, Table 1). One of these samples was both HHV6 and EV IgM positive, but clinically the case resembled more an HHV6 infection.

### Age distribution

**Figure 1:**
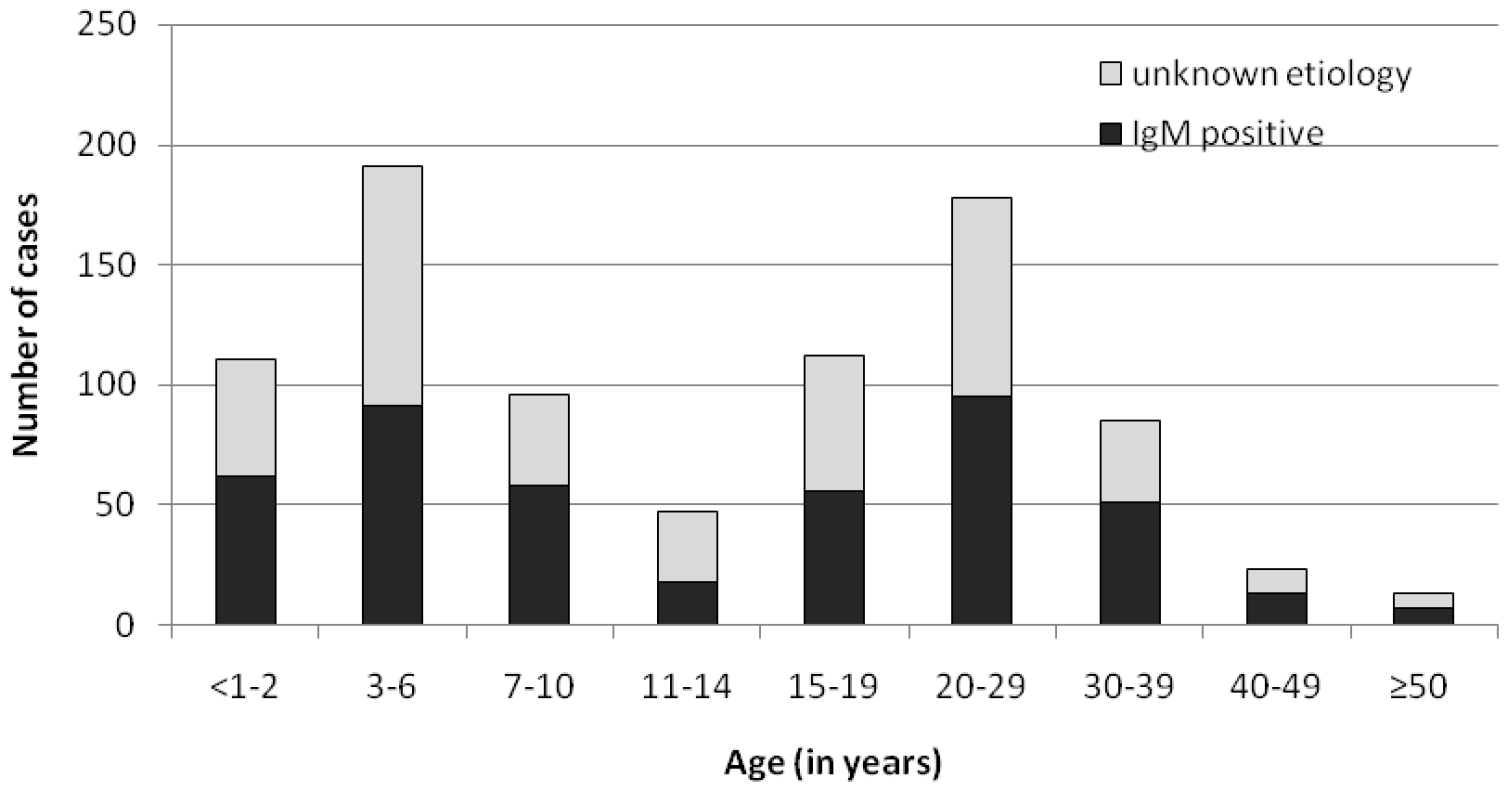
Numbers of samples positive for IgM antibodies against any of the viruses tested according to age groups, Belarus, 2009–2011.

About half of the patients tested (52.0%) were less than 15 years old. The largest age groups were the 3–6 and 20–29 year-olds (Figure 1). In each age group between 50 and 60% of the patients were diagnosed except for the 11–14 years bracket (39.6%) (Figure 1).

**Figure 2:**
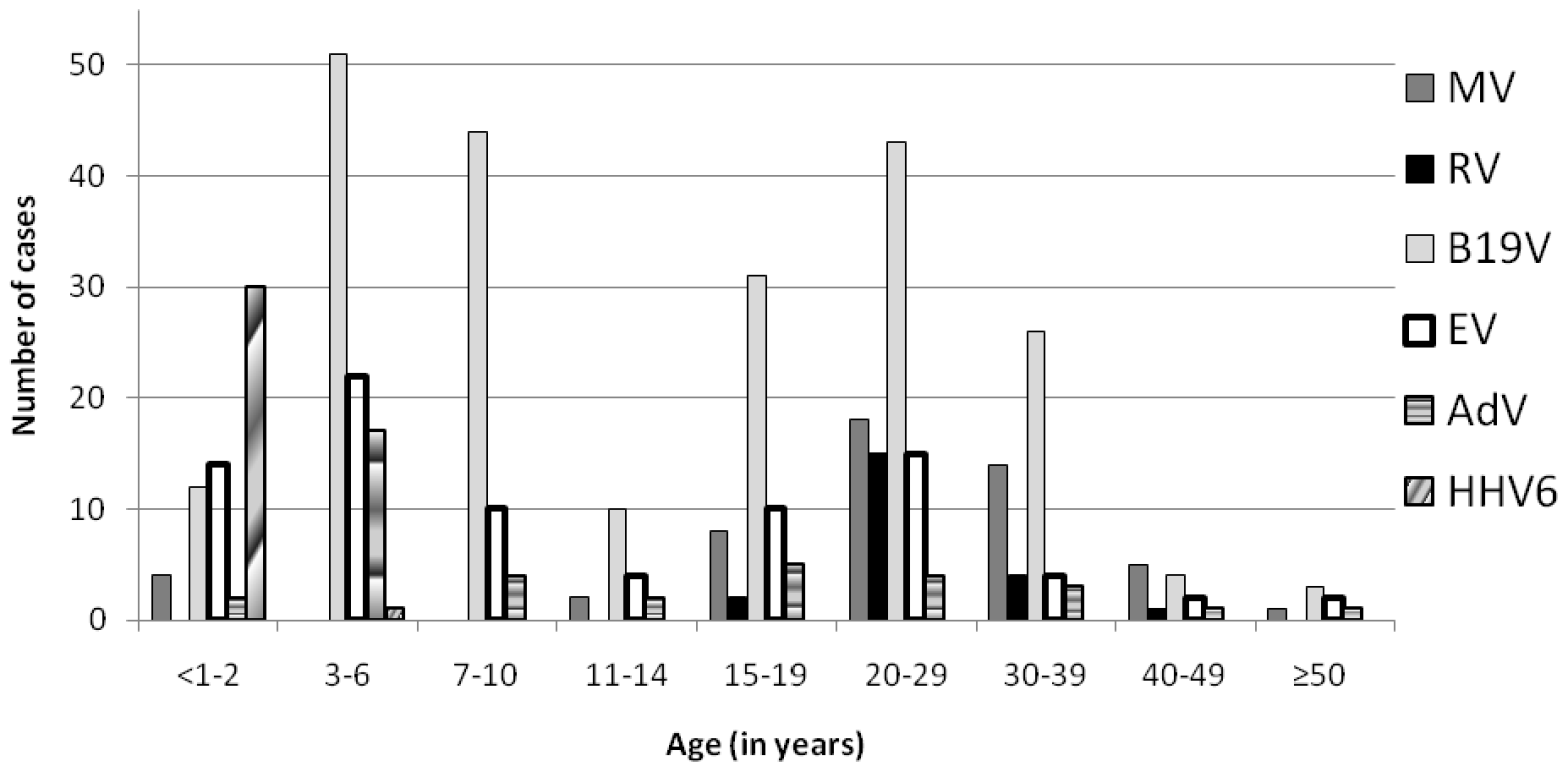
Numbers of samples positive for specific IgM antibodies against MV, RV, B19V, EV, AdV and HHV6 in different age groups.

The frequency of the viruses differed between age groups (Figure 2). 88.5% (46/52) of the measles patients were at least 15 years old and 61.5% (32/52) of the patients were 20 to 39 years old. Only 6 MV positive samples were from children. The 22 rubella positive sera were from 16–44 year-old patients.

B19V, EV and AdV infections were found both in children and adults. 52.2% (117/224) of the B19V-positive samples were from 2–14 year-olds. 3–10 year-old children and adults between 20 and 29 years were most affected (Figure 2). B19V was the main etiology in all age groups above 3 except for the 40–49 years age bracket during a measles outbreak.

EV infections were most common in children up to 6 years of age, but also in 20–29 year-old adults. AdV infections were confirmed mainly in 3–6 year-old children with only rare cases in other age groups.

In children <3 years old, HHV6 dominated and was responsible for 48.4% of the positives in this age group. Among the 31 children positive for HHV6, 20 (64.5%) were 6–11 months old, 6 were 1 year old, 4 were 2 years old and a single HHV6 patient was 3 years old.

### Seasonality

**Figure 3:**
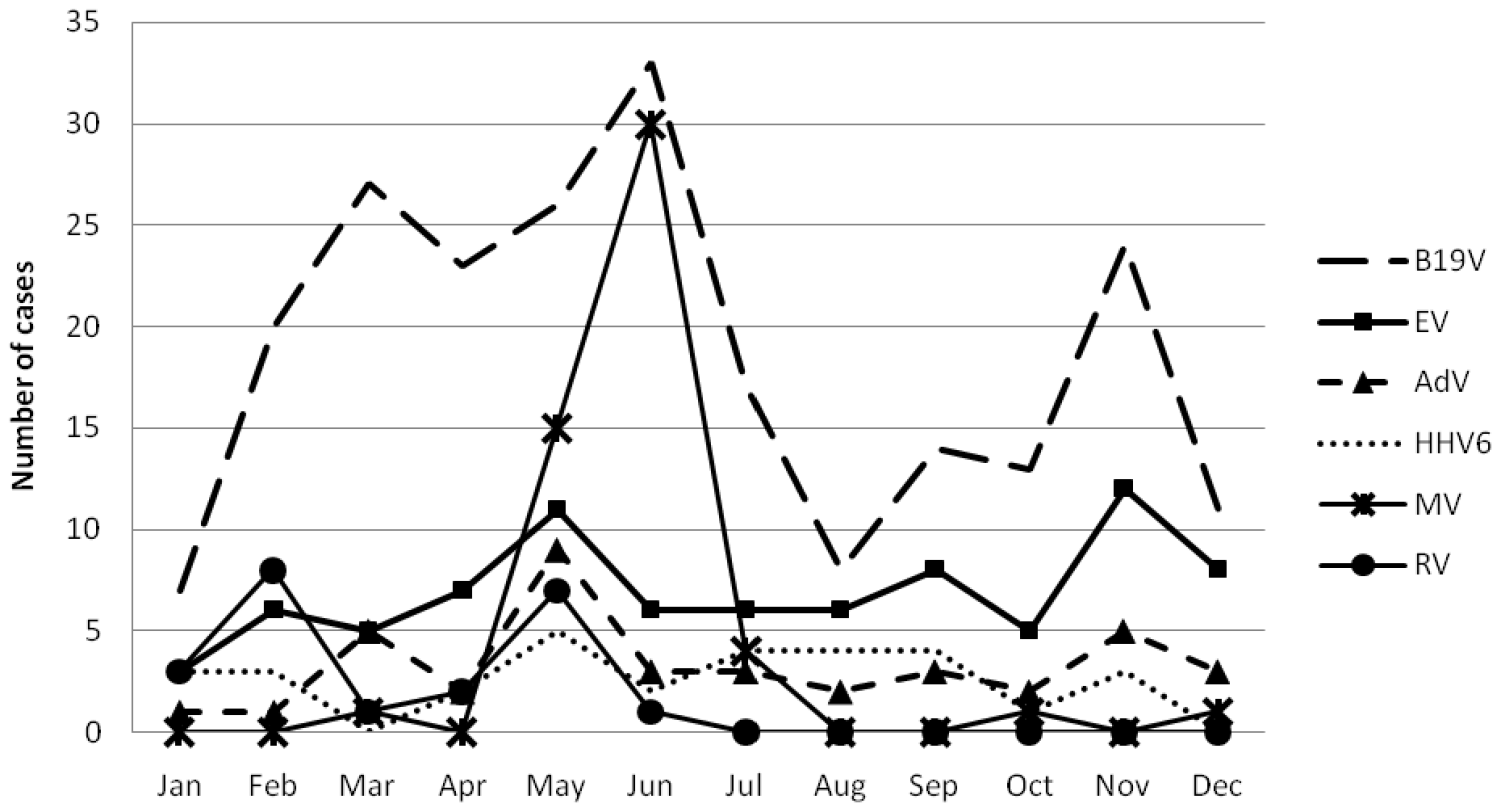
Distribution of MV, RV, B19V, EV, AdV and HHV6 IgM positive samples by months, 2009–2011.

The increase in both measles and rubella incidence was due to limited outbreaks in 2011. Almost all rubella cases (20/22, 90.1%) were reported between January and June 2011 and 94.2% of the measles cases were recorded between May and July 2011 (Figure 3). B19V infections were found throughout the year with peak incidences in March, June and November. Also EV, AdV and HHV6 cases occurred essentially throughout the year (Figure 3).

## Discussion

As a result of successful vaccination strategies, the measles and rubella incidence has decreased dramatically in Belarus. Between 2009 and 2011, 80 to 99% of all suspected cases were negative for measles- and rubella-specific IgM antibodies. Similar rates were reported from other countries in the final stages of disease elimination [4], [5], [7]–[9]. Most suspected cases were reported among the 3–6 and 20–29 year-olds, possibly reflecting more contacts with different agents in nursery schools for the former, and more and closer contacts with small children in young adults. Also greater attention to exanthematous diseases in women of childbearing age could play a role.

Between 190 and 390 measles/rubella suspected cases were reported and investigated every year and at least 2 cases per 100 000 population were thus discarded as measles/rubella double negatives. Most suspected cases were reported in 2011 when a limited spread of both measles and rubella occurred after years with single or no cases. During the same year the measles incidence was also higher in many other European countries [10]. In Belarus two single cases of genotype D4 were imported from Germany and a limited outbreak was caused by a genotype D8 virus of unknown origin [11]. Half of the cases were either not vaccinated against measles, had received one dose of vaccine or had an unknown vaccination status. The patients vaccinated with two doses were mostly between 16 and 30 years old. A similar observation had been made in the Ukraine and was linked to cold-chain failures and the use of a more thermo-labile vaccine [12]. Similar explanations may apply to Belarus, although secondary vaccine failures and waning of antibodies may have also played a role. Rubella was imported to Belarus from Vietnam where a large outbreak was registered at the time [13] and initially affected Vietnamese temporary workers before causing a limited outbreak among resident people in Minsk city. In contrast to measles, 91% of all rubella patients were unvaccinated and none had received two doses of vaccine.

More than 50% of the suspected measles/rubella infections were caused by any of the 6 viruses investigated, but only less than 9% were due to measles or rubella. Thus the panel of the four additional assays is suitable to complement the routine MV and RV testing in Belarus. The same 4 viruses were also frequent in measles/rubella negative children with rash/fever in Finland, where they accounted for about 37% of all cases [4]. In Brazil, testing for all 6 viruses plus 4 additional pathogens allowed to diagnose about 42% of the rash/fever cases [5].

B19V caused 26% of all cases or about 60% of all diagnosed cases and was the main reason of measles/rubella suspected diseases in each of the three consecutive years. Between 2005 and 2008, B19V accounted for nearly 36% of the measles/rubella double negative cases in Belarus [14]. Therefore all measles/rubella double negative samples are now also tested for B19V, providing a diagnosis to about 3 times more patients and their doctors (8.6% MV+RV compared to 26.2% B19V). The importance of B19V was also shown in studies from Argentina, Finland, England, and Poland where it was confirmed in 17% to 40% of suspected measles/rubella cases [4], [7], [8], [15].

Similar to studies in children from Finland [4] and England [7], we found that about 10% and 5% of the rash cases (or 15% and 7% of the cases tested) were caused by EV and AdV infection, respectively. Acute AdV infection is mainly diagnosed by detecting a significant rise of IgG antibodies in paired sera or by viral culture [4], [7], but no such samples were available for the present investigation. Therefore, we screened the sera for specific IgM antibodies against AdV, similar to the approach used in previous studies [16]–[18].

In Finland, England and the Caribbean, HHV6 infection was detected in 3–7% of all exanthemas [4], [7], [19]. In a study from Brazil, HHV6 was with 25% even the most common etiology of all rash/fever cases [5]. About one quarter of Thai rash/fever patients up to 14 years were HHV6 positive [20]. In our study, HHV6 was tested only in children up to 3 years of age and it was the main agent in the less than 1 to 2 year-olds where it caused about 20% of all cases or 48% of the diagnosed cases. In Brazil, the peak incidence was observed among 6–11 months-old infants and 75% of all HHV6 cases occurred between 6 and 17 months of age [6]. Until now, HHV6 infection is not part of the routine diagnosis in Belarus, but our data suggest that at least samples from measles/rubella suspected children below 24 months should be tested for this virus.

In conclusion, the results of the present study showed that MV, RV, B19V, EV, AdV and HHV6 accounted for more than half of all measles/rubella suspected cases in Belarus. Since Belarus has entered the final stages of measles and rubella elimination, B19V is responsible for most suspected cases in the country. Laboratory investigation of measles/rubella double negative specimens for B19V, EV, AdV and at least in children up to 2 years also for HHV6 can help to further strengthen rash/fever surveillance in Belarus providing a diagnosis for more than 5 times as many patients as measles/rubella testing alone. Testing for these additional pathogens should also be considered in other countries with a similar geographical location and measles/rubella control stage.
